# Early detection of pancreatic cancer by liquid biopsy “PANLIPSY”: a french nation-wide study project

**DOI:** 10.1186/s12885-024-12463-8

**Published:** 2024-06-10

**Authors:** Thomas Bardol, Antoine M. Dujon, Valerie Taly, Catherine Dunyach-Remy, Jean-Philippe Lavigne, Bruno Costa-Silva, Keerthi Kurma, Zahra Eslami-S, Laure Cayrefourcq, Cindy Canivet, Fabrice Muscari, Barbara Bournet, Catherine Alix-Panabières

**Affiliations:** 1 Laboratory of Rare Circulating Human Cells – Liquid Biopsy Lab, Institut Universitaire de Recherche Clinique (IURC), University Medical Center of Montpellier, 641, Avenue du Doyen Gaston Giraud, Cedex 5 34093 Montpellier, France; 2 CREEC/CANECEV MIVEGEC (CREES), University of Montpellier, CNRS, Montpellier, IRD France; 3 European Liquid Biopsy Society (ELBS), Hamburg, Germany; 4 School of Life and Environmental Sciences, Deakin University, Waurn Ponds, Victoria, Australia; 5 Équipe Labélisée Ligue Nationale Contre Le Cancer, Centre de Recherche Des Cordeliers, Université Paris Cité, UMR-S1138, CNRS SNC5096, Paris, France; 6 METHYS Dx, 67 Rue Saint-Jacques, Paris, France; 7 Department of Microbiology and Hospital Hygiene Bacterial Virulence and Chronic Infections, University of Montpellier CHU Nîmes, INSERM U1047, Nîmes, France; 8 Champalimaud Physiology and Cancer Programme, Champalimaud Foundation, 1400-038 Lisbon, Portugal; 9 Department of Gastroenterology and Pancreatology, CHU - Rangueil and the University of Toulouse, 1 Avenue Jean Poulhès, Cedex 9 50032, 31059 Toulouse, TSA France; 10 Digestive Surgery and Liver Transplantation Department, Toulouse University Hospital, Toulouse, France

**Keywords:** Liquid biopsy, Pancreatic cancer, Circulating tumor cells, Circulating tumor DNA, Exosomes, Multi-omic approach

## Abstract

**Background:**

Pancreatic cancer, predominantly characterized by ductal adenocarcinoma (PDAC) accounts for 90% of cases and is the fourth leading cause of cancer-related deaths globally. Its incidence is notably increasing. This poor prognosis is primarily due to late-stage diagnosis (approximately 70% to 80% of patients are diagnosed at an advanced stage), aggressive tumor biology, and low sensitivity to chemotherapy. Consequently, it is crucial to identify and develop a simple, feasible and reproducible blood-based signature (i.e., combination of biomarkers) for early detection of PDAC.

**Methods:**

The PANLIPSY study is a multi-center, non-interventional prospective clinical trial designed to achieve early detection of PDAC with high specificity and sensitivity, using a combinatorial approach in blood samples. These samples are collected from patients with resectable, borderline or locally advanced, and metastatic stage PDAC within the framework of the French Biological and Clinical Database for PDAC cohort (BACAP 2). All partners of the BACAP consortium are eligible to participate. The study will include 215 PDAC patients, plus 25 patients with benign pancreatic conditions from the PAncreatic Disease Cohort of TOuLouse (PACTOL) cohort, and 115 healthy controls, totaling 355 individuals. Circulating biomarkers will be collected in a total volume of 50 mL of blood, divided into one CellSave tube (10 mL), two CELL-FREE DNA BCT® preservative tubes (18 mL), and five EDTA tubes (22 mL in total). Samples preparation will adhere to the guidelines of the *European Liquid Biopsy Society* (ELBS). A unique feature of the study is the AI-based comparison of these complementary *liquid biopsy* biomarkers.

Main end-points: *i)* to define a liquid biopsy signature that includes the most relevant circulating biomarkers, *ii)* to validate the multi-marker panel in an independent cohort of healthy controls and patients, with resectable PDAC, and *iii)* to establish a unique liquid biopsy biobank for PDAC study.

**Discussion:**

The PANLIPSY study is a unique prospective non-interventional clinical trial that brings together liquid biopsy experts. The aim is to develop a biological signature for the early detection of PDAC based on AI-assisted detection of circulating biomarkers in blood samples (CTCs, ctDNA, EVs, circulating immune system, circulating cell-free nucleosomes, proteins, and microbiota).

**Trial registration:**

ClinicalTrials.gov Identifier: NCT06128343 / NCT05824403. Registration dates: June 8,2023 and April 21, 2023.

## Background

Pancreatic cancer (PC) is currently the fourth leading cause of cancer-related deaths worldwide. Over the past two decades, its prevalence has steadily risen, particularly in Europe, with 150,000 new cases reported in 2018 and 95,000 deaths per year (Global Cancer Observatory (iarc.fr)). Moreover, PC has the worst prognosis of any cancer type in Europe, with a median survival time of 4.6 months. Consequently, it is projected that by 2030, PC will become the second leading cause of cancer-related deaths in Europe and globally. In France, the incidence of PC increased from 7,500 cases per year in 2009 to over 14,000 per year in 2018, with 11,456 deaths reported in 2018 (5-year overall survival rate: 11%). This poor prognosis can be attributed to the late-stage diagnosis of PC in approximately 70%-80% of patients (~ 50% of patients are diagnosed with stage IV PC), its aggressive biology, and the partial response to currently available chemotherapies. Furthermore, there is no effective screening program due to the absence of a biological test for early PC detection. Therefore, the most significant challenge in managing PC is early detection, especially in patients where PC has not developed yet, such as patients at risk (PAR) and patients with pre-neoplastic cystic lesion of the pancreas (PPNL), such as intraductal papillary mucinous neoplasms. Despite promising studies on early PC biomarkers, particularly in risk groups (PAR, PPNL), no biomarker suitable for clinical diagnosis has been identified [1]. Carbohydrate antigen 19–9 (CA19-9) is the most commonly used and evaluated blood-based PC biomarker; however, its specificity is limited as elevated CA19-9 levels are also associated with other clinical conditions [2]. In this context, it is critical to identify and develop a simple, feasible, and reproducible biomarker.

Currently, pancreatic ductal adenocarcinoma (PDAC; the most common histological form of PC) is diagnosed using tissue biopsies (from the primary tumor or from secondary lesions) [3]. Tissue biopsies are typically performed with endoscopic (for resectable, borderline, or locally advanced primary tumor lesions), or radiological guidance (for metastatic lesions or when endoscopy is contra-indicated). Endoscopy-guided biopsies are often technically challenging to perform, making them operator-dependent and occasionally ineffective. In the worst-case scenario, a single patient may undergo repeated invasive procedures without receiving a definitive diagnosis. Complications of endoscopy-guided biopsy include bleeding, duodenal perforation, and more commonly acute pancreatitis. Severe acute pancreatitis may delay the tumor surgical removal or postpone a new tissue biopsy, if the previous biopsy was not contributive. In this context, it is crucial to develop an alternative method to tissue biopsies for PC diagnosis. Lastly, despite advances in radiological work-up (thoraco-abdomino-pelvic CT and liver MRI), patients with upfront resectable PC frequently experience early post-operative progression, most likely due to the failure to detect infra-clinic metastatic disease. Therefore, to improve the prognosis of patients with PC, new biomarkers are required for earlier, pre-symptomatic diagnosis.

Blood-based biomarkers, such as circulating tumor cells (CTCs), circulating cell-free tumor DNA (ctDNA), extracellular vesicles (EVs), circulating cell-free nucleosomes (cfnucleosomes), circulating cell-free proteins, circulating immune cells (CIC), and circulating microbiota, may serve as potential indicators of tumor burden in patients with cancer. These minimally invasive "*liquid biopsies*” (*LB*) have garnered significant attention due to their clear clinical implications for personalized medicine, such as the identification and stratification of patients with cancer. *LB* were introduced as a new diagnostic concept in 2010 [4] and offer several clinical applications [5]. *LB* methods based on ctDNA methylation patterns for the early diagnosis of PC have shown promising results [6]; also, the detection of *KRAS*-mutated ctDNA was correlated to post-operative recurrence [7]. However, these *LB* approaches based on a single analyte might have a low positive predictive value and might not be sufficient for screening purposes [8, 9]. We believe that early cancer detection by *LB* needs to rely on a combination of appropriate circulating biomarkers to obtain a precise cancer profile in real-time [10]. *LB* use for the early detection of PDAC, which represents 90% of histologically proven PC, is of high public interest, but it faces serious challenges concerning the specificity and sensitivity of the current *LB*-based assays. Therefore, in this study we propose a systematic approach with the aim of assessing different, recently developed complementary *LB* biomarkers (CTCs, ctDNA, EVs, cfnucleosomes, proteins, CIC, and circulating microbiota) as a novel noninvasive method for the early detection of resectable PDAC. The main hypothesis of the PANLIPSY study is that the *LB* signature (combination of circulating biomarkers), identified using AI-based algorithms, may allow the detection of early-stage PDAC in blood samples from patients with pancreatic cancer and benign pancreatic lesions (prospective clinical trials BACAP 2 and PACTOL). This could improve the detection of PDAC at an early stage to enhance patients’ oncological outcomes.

## Methods and design

### Aims of PANLIPSY

The main objective of this non-interventional prospective clinical trial is to define a *liquid biopsy (LB)* signature that includes the most relevant analytes currently available: CTCs, ctDNA, circulating nucleosomes, circulating proteins, EVs, CICs, and circulating microbiota in blood samples from patients with histologically confirmed PDAC (all stages) compared to healthy controls (HCs) and patients with benign pancreatic diseases. This signature will combine the results of all *LB* analytes into a multi-marker panel, designed through computational combinatorial analysis with AI-based algorithmic approaches. In the second part of the study, this multi-marker panel will be validated in an independent cohort of HCs and patients with resectable PDAC. The project will also establish a unique liquid biopsy bio-bank for the PDAC study.

### Study population

A total of 215 patients with PDAC, 25 patients with benign pancreatic conditions (BPC) and 115 age- and sex-matched HCs will be included during the study period.

### Pancreatic ductal adenocarcinoma patients (BACAP 2 prospective clinical trial / NCT06128343)

The collection of all biological samples from patients with PDAC will be supervised by the BACAP consortium, which was developed to provide the scientific community with biological material to identify new markers, including diagnostic and prognostic biomarkers [11].

### Inclusion criteria

- Age 18 years or older.
- Patients with cytologically and/or histologically confirmed pancreatic ductal adenocarcinoma, regardless of the cancer stage (resectable, borderline or locally advanced or metastatic).

### Exclusion criteria

- Patients who have already initiated treatment for pancreatic cancer
- Other cancers (excluding prior pancreatic cancer or skin cancer other than melanoma) within 5 years from eligibility screening.
- Pregnant or nursing woman, or women of childbearing age not willing to use contraception
- Protected and vulnerable adults
- Individuals not covered by health insurance
- Patients unable to understand and sign written informed consent.

### Pancreatic benign lesions (PACTOL prospective clinical trial / NCT05824403)

PACTOL is a prospective cohort dedicated to pancreatic pathologies other than cancer. Its aim is to develop tools for positive or differential diagnosis between potentially malignant and benign pancreatic pathologies.

### Inclusion criteria:

- Age 18 years or older
- Patients with inflammatory or cystic exocrine pancreatic pathology.

### Exclusion criteria:

- Patients with pancreatic cancer pathology
- Other cancers (excluding prior pancreatic cancer or skin cancer other than melanoma) within 5 years from eligibility screening
- Pregnant or nursing woman, or women of childbearing age not willing to use contraception
- Protected and vulnerable adults
- Individuals not covered by health insurance
- Patients unable to understand and sign written informed consent.

#### Healthy controls

After the completion of patients’ inclusion in each phase, the INOVIE Biobank (INOSPECIMENS) will graciously provide blood samples from age- and sex-matched HCs (*n* = 85 at the end of the discovery phase then *n* = 30 for the validation phase).

#### Sample size justification

DISCOVERY PHASE (85 patients with resectable PDAC and 85 HCs):

With a total sample size of 170 (*n* = 85 patients with resectable PDAC and *n* = 85 HCs), a two-sided 95% confidence level with an AUC = 0.85, produces a confidence interval width of 0.118 (distance from AUC to the limit = 0.059; nQuery software / Confidence interval for AUC).

VALIDATION PHASE (30 patients with resectable PDAC and 30 HCs):

The sample size is based on the recruitment potential from the BACAP cohort for a 12-month period of inclusion and corresponds to ~ 30% of the total sample.

### Design

In this multi-center non-interventional prospective study, different blood sample-based assay formats will be evaluated as tools for early PDAC detection. Patients with cytologically and/or histologically confirmed PDAC will be included and classified as resectable, borderline or locally advanced, and metastatic according to tumor staging (cancer cohort). Patients with BPC and HCs will also be enrolled as non-cancer cohorts. All samples from patients with PDAC (*n* = 215) will be collected thanks to the BACAP 2 prospective clinical trial. Patients will be treated according to the current standard of care. Patients with BPCBPC will be collected through the PACTOL cohort. The total number of included patients with PDAC, BPC and HC will reach 355 (DISCOVERY PHASE: *n* = 295; VALIDATION PHASE: *n* = 60). The study work plan is summarized in Table 1.

**Table 1:** Synthetic description of the work plan at the work package level of PANLIPSY study protocol

WP	Brief Task Description	Participant(s) (team leader)
**WP1: Patients recruitment/inclusion (*n* = 355)** Leaders: Pr BOURNET	DISCOVERY PHASE (*n* = 295)	BACAP 2-PACTOL (B.B; F.M)/INOVIE Biobank (Christophe BENA)
	VALIDATION PHASE (*n* = 60)	BACAP 2 (B.B; F.M)/INOVIE Biobank (Christophe BENA)
**WP2: Liquid Biopsy Biomarkers** Leader: Pr ALIX-PANABIÈRES	CTC detection and characterization: CellSearch® system	LCCRH – Liquid Biopsy Lab (C.A-P)
	ctDNA	MEPPOT team (VT)/LCCRH – Liquid Biopsy Lab (C.A-P)
	EVs	LCCRH – Liquid Biopsy Lab (C.A-P)
	Circulating cell-free nucleosomes	LCCRH – Liquid Biopsy Lab (C.A-P)
	Circulating proteins	LCCRH – Liquid Biopsy Lab (C.A-P)
	CIC	LCCRH – Liquid Biopsy Lab (C.A-P)
	Circulating microbiota	INSERM U1047 (C.R; J-P.L)/LCCRH – Liquid Biopsy Lab (C.A-P)
	Biobanking (cells/plasma)	LCCRH – Liquid Biopsy Lab (C.A-P)
**WP3: Artificial intelligence-based algorithm** Leader: Dr DUJON	DISCOVERY PHASE results analysis	CREEC/CANEVEC (A.D)
	VALIDATION PHASE results analysis	CREEC/CANEVEC (A.D)

**Fig. 1:**
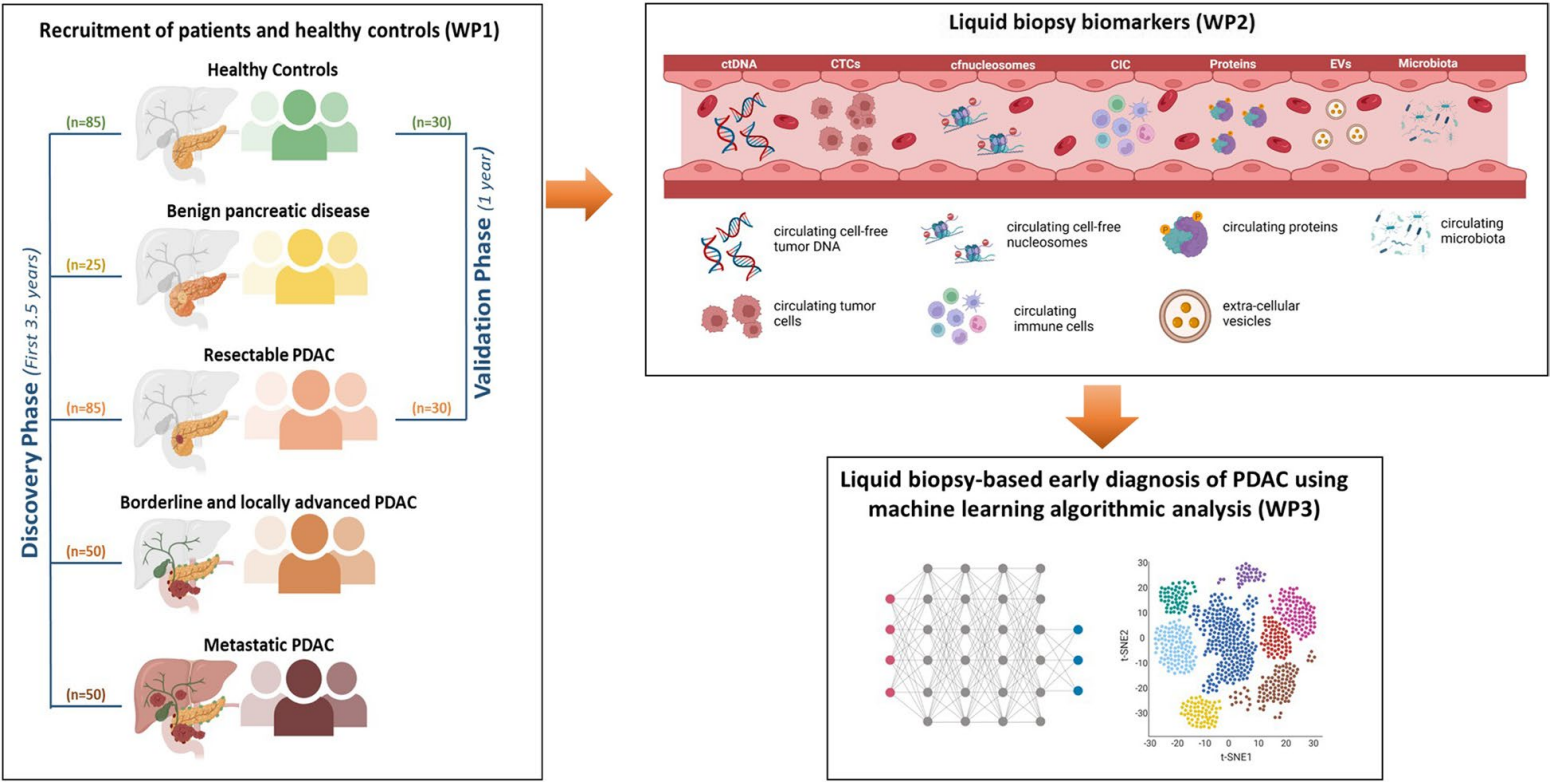
Workflow of the PANLIPSY study. The PANLIPSY study is structured around three work packages. Work package 1: recruitment of PDAC patients, patients with benign pancreatic conditions and HCs; Work package 2: liquid biopsy multi-omic approach; Work package 3: Artificial intelligence-based algorithm analysis

The PANLIPSY study unites a unique team of experienced clinical centers of excellence to collect blood samples from patients with PDAC, BPC, and HCs. These samples will be used for *LB* biomarker analysis (CTCs, ctDNA, EVs, cfnucleosomes, circulating proteins, CIC, and circulating microbiota). To develop the multi-marker *LB* signature, we will employ deep learning methodologies to devise an algorithm that identifies the optimal combination of blood markers (Fig. 1).

PANLIPSY is a five-year project divided into two distinct phases: a discovery (3.5 years) and a validation phase (1.5 years), as represented in Fig. 1.

### Discovery phase

During the first 3.5 years, patients with resectable (*n* = 85), borderline or locally advanced stage (*n* = 50), metastatic PDAC (*n* = 50), and BPC (*n* = 25) will be included. Additionally, 85 age- and sex-matched HCs will be recruited at the end of this period (in-kind contribution of INOSPECIMENS from the Groupe INOVIE). All circulating biomarkers listed above will be detected in patients’ blood samples. The exploratory design is essential to precisely determine the best *LB* signature based on the cancer stage. Patients with BPC and HCs (control groups) will also be included to eliminate potential confounding markers, background noise, and misleading biological signatures. This discovery approach will also provide different *LB* signatures correlated with PDAC aggressiveness.

### Validation phase

Secondly, the defined combination of candidate biomarkers will be validated in the final year by blindly classifying ‘patients with early-stage/resectable PDAC (*n* = 30)’ from ‘HCs’ (*n* = 30).

### Data collection and follow-up

Data are collected at the patient’s inclusion, during the diagnosis process, and before any cancer treatment administration (upfront surgery, chemotherapy-radiochemotherapy, and/or surgery and/or best supportive care), and through follow-up until death. As previously defined by the BACAP protocol, clinical data collected will include: disease presentation, demographic data, baseline biology, radiology, endoscopic ultrasound, tumor staging, pathology reports, treatments, follow-up (including biological and radiological), and survival [11]. Patient follow up will be managed according to standard-of-care and will depend on the practices of each center. All these data will be collected and stored through an e-observation system at a centralized data center.

### Liquid biopsy assays

**Fig. 2:**
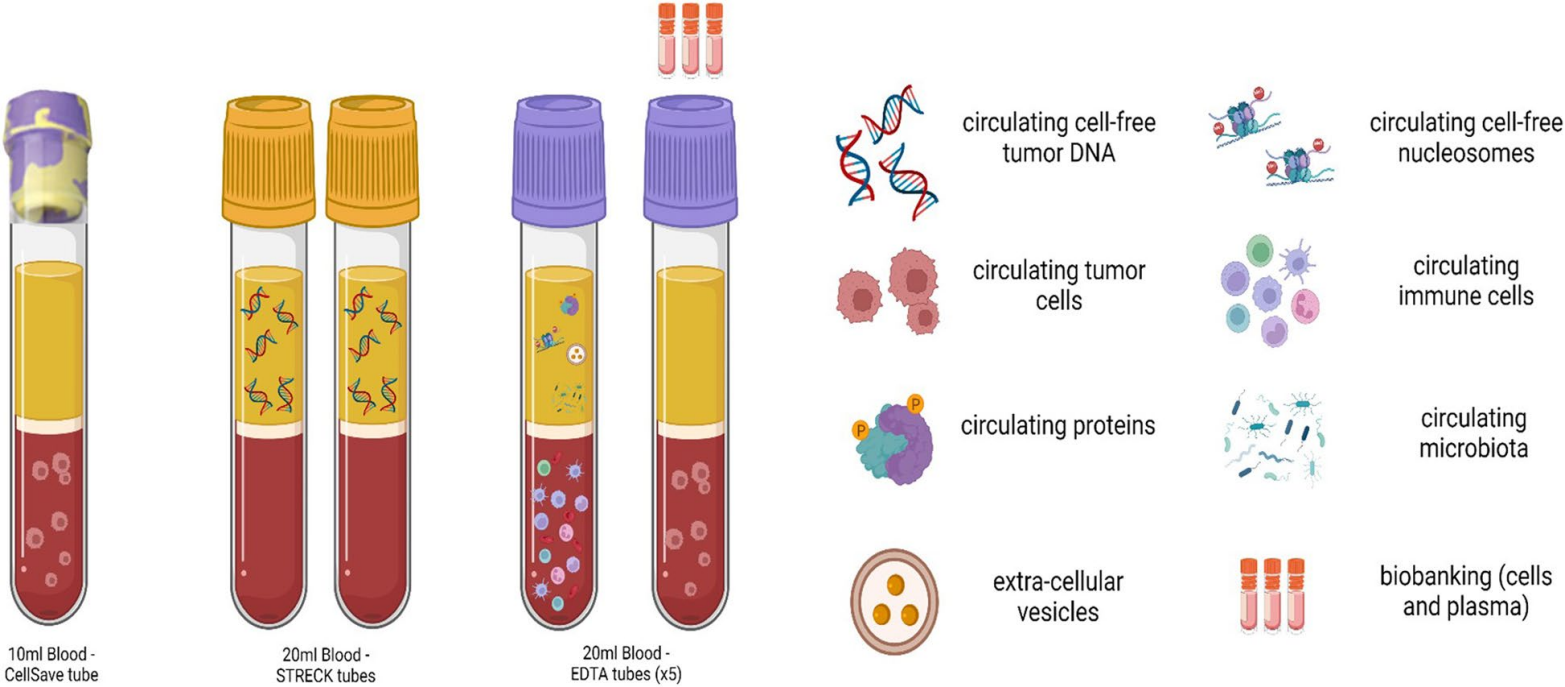
Blood collection. 50 mL of peripheral blood will be drawn and collected in eight tubes: one CellSave® tube (10 mL) for CTC detection, two CELL-FREE DNA BCT® preservative tubes (Streck™, 18 mL in total) for ctDNA analysis, and five EDTA tubes (22 mL in total) for the detection of extracellular vesicles, circulating cell-free nucleosomes, circulating proteins, circulating microbiota and biobanking

In total, 50 mL of blood will be collected in different tubes for LB assays: one CellSave® tube (10 mL), two CELL-FREE DNA BCT® preservative tubes (Streck™, 18 mL in total), and five EDTA tubes (22 mL in total) (Fig. 2).

Blood sample management will be centralized at the Laboratory LCCRH – Liquid Biopsy Lab, University Medical Center of Montpellier. Whole blood components: plasma, cells and platelets, will be isolated from blood tubes. Samples, prepared following the European Liquid Biopsy Society (ELBS) guidelines (elbs.eu), will then be shipped to the different partners for *LB* analysis. Biological samples are collected and handled following a unique protocol to ensure the homogeneity of the biological resource.

### CTC detection and characterization

Among the various CTC technologies, the CellSearch® system (MENARINI) has gained considerable attention over the past years. It is the first CTC assay cleared by the US FDA and currently the gold standard for CTC detection, and the most widely used technology for prognostic purposes in clinical studies. It is based on the enumeration of epithelial cells that are separated from whole blood samples by positive enrichment using anti-EpCAM antibodies coated with magnetic beads. Specifically, the ferrofluid reagent consists of particles with a magnetic core surrounded by a polymeric layer coated with anti-EpCAM antibodies for directly capturing CTCs. After immunomagnetic capture and enrichment, fluorescent antibodies (against cytokeratin, panCK (CK8/18/19) that are expressed in the CTC cytoplasm; and against CD45 expressed at the leukocyte surface) and the nuclear dye DAPI are added for CTC identification and enumeration. CTCs are defined as EpCAM^+^/panCK^+^/DAPI^+^/CD45^−^ cells. The 4th channel allows assessing an additional marker to characterize directly CTCs at the single-cell level.

### Circulating tumor DNA

The 18 mL whole blood, collected in CELL-FREE DNA BCT® preservative tubes (Streck™, see above) will be processed within 7 days. The following steps will be performed: *(i)* centrifugation at 1600 g/10 min/18–20 °C, *(ii)* separation of plasma from buffy coat, *(iii)* plasma centrifugation at 16000 g/10 min/18–20 °C. Plasma and buffy coat will be immediately stored at -80 °C. Before cell-free DNA extraction, the sample will be defrosted at room temperature and submitted to a second centrifugation for 15 min at 2700 g. Circulating cell-free DNA (ccfDNA) will be extracted using the QIAamp® Circulating Nucleic Acid Kit (QIAGEN®, Germany) according to the manufacturer’s instructions with an elution volume of 50 µL (AVE buffer). Incubation with proteinase K will be performed for 30 min at 60 °C. Each DNA extraction batch included a blank sample (Phosphate-buffered saline, PBS). DNAs will be quantify using Qubit™ dsDNA HS Assay Kit (Invitrogen™, Waltham, MA, USA) and then immediately stored at -20 °C.

20 µL of ccfDNA extracted from plasma will be bisulfite-converted using the EZ DNA Methylation-lightning Kit (ZYMO Research), according to the manufacturer’s instructions. Bisulfite-converted ccfDNA will be eluted in M-elution buffer. Preferentially, bisulfite-converted DNA will be immediately tested by ddPCR or when needed, stored up to one week at -20 °C until ddPCR. Three controls will be used in each conversion batch, the blank PBS sample mentioned above, 10 ng human genomic DNA extracted from whole blood (pool from multiple donors, Promega) as negative control and 10 ng enzymatically universally methylated human genomic DNA (ZYMO Research, USA) as positive control. The DNA concentrations of the control DNA samples will be determined at reception using the Qubit™ DNA HS Assay Kit (Invitrogen™).

All plasma samples will be screened for the presence of methylation markers by droplet-based digital PCR (Met-ddPCR) as previously described [12]. The assay will target the detection of hypermethylation of HOXD8 and POU4F1 genes as well as a methylation-insensitive target on the albumin (ALB) gene used as an internal control of the amount of DNA analyzed in each PCR well [13]. All ddPCR assays will be performed according to the updated dMIQE guidelines [14].

### Extracellular vesicles

EVs purification/isolation from plasma will be performed using sequential steps of centrifugation and ultracentrifugation. EVs will be characterized by nanoparticle tracking analysis (NTA) using a NanoSight NS300 equipped with a red laser (638 nm) to determine particle concentration and size distribution (Malvern Panalytical, United Kingdom) and with the NTA v3.4 software, particle concentration and size distribution will be analyzed. The total protein content of EV samples will be determined using the Pierce™ BCA Protein Assay Kit (Catalog number: 23225; Thermo Fisher Scientific).

Analysis of IgG^+^ EV population: vesicle flow cytometry analyses will be performed using an anti-human IgG F(ab’)2 conjugated with Fluor 647 antibody (Abbexa abx142503, Houston, TX, United States). Subsequently, a labelling with Carboxyfluorescein Diacetate Succinimidyl Ester (CFSE – ThermoFisher Scientific LTI C34554, MA, United States) will be performed to evaluate the vesicularity of the samples. Labelled samples will then be analyzed with the flow cytometer Apogee A60-Micro-Plus (Apogee Flow Systems, United Kingdom). The acquired data will be exported and analyzed with FlowJo software v10.4.2 (FlowJo LLC, United States).

### Circulating cell-free nucleosomes

Circulating cell-free nucleosome structures will be measured using quantitative Nu.Q® Immunoassays (Belgian Volition SRL, Namur, Belgium) performed according to the manufacturer’s instructions, as reported previously [15, 16]. Briefly, the assays consist of a sandwich immunoassay which employs a capture antibody directed against a specific histone modification or variant, coupled to magnetic beads and an acridinium ester labeled anti-nucleosome detection antibody.

Each Nu.Q® immunoassays will be performed on the IDS-i10 automated immuno-analyzer system (Immunodiagnostic Systems Ltd (IDS), UK). 50 µL of plasma samples will be incubated with the detection antibody. Then, the magnetic particles beads coated with a monoclonal anti-histone modification capture antibody will be added. Finally, after a wash step, trigger solutions will be added and the light emitted will be measured by a luminometer. Results will be expressed in Relative Light Unit (RLU), and the concentrations will be evaluated using a four-parameter logistic regression of a reference standard curve. Samples will be analyzed in duplicate.

### Circulating proteins

O-link PEA is an immunoassay-based technology that relies on matched antibody pairs for dual recognition of proteins (biomarker) in biological samples. The two matched antibodies, which target the same protein, are conjugated to unique DNA oligonucleotides with a stretch of sequences complementary to each other. When the antibody pair binds to the target protein, complementary DNA oligonucleotides hybridize, and get extended and amplified by DNA polymerase. Each amplified DNA pair contains unique barcode sequences that are read using qPCR. The library includes 3000 highly validated protein assays that cover all major biological pathways.

### Circulating immune cells

CIC analyses will be performed using enriched peripheral blood mononuclear cells (PBMCs) stained for multi-parametric FACS analyses. PBMCs will be analyzed to define and characterize various immune cell populations and to determine the expression profiles of T-cells, cytotoxic T-cells, T-regulatory cells (T-regs), natural killer (NK) cells, B-cells and immune checkpoint inhibitor. A second tube will be used to determine the monocytes/macrophages expression profile. The Zombie red™ Fixable Viability kit will be used to exclude dead cells. Samples will be analyzed by Beckman Coulter Gallios Flow Cytometry, and data will be collected with the Kaluza acquisition software and analyzed with the Kaluza software.

### Circulating microbiota

The number of 16S rRNA gene copies, which reflect the circulating DNA microbiota, will be measured in plasma by qPCR following the protocol previously published [17]. DNA will be extracted from 200 μL of plasma with the QIAcube® automatic extractor (Qiagen, Courtaboeuf, France) and the QIAamp MinElute ccfDNA® kit (Qiagen, Courtaboeuf, France) as per the manufacturer's instructions. The amplified region will have a size of 199 bp, and the V5 region of the 16S rRNA gene will be targeted. The 16S rRNA gene real-time PCR will be performed twice on two biological duplicates for each sample. A negative control using molecular biology grade pure water will be included. A standard curve will be created from serial dilutions of synthetic DNA containing known copy numbers of the template. The assays will be conducted using a LightCycler 480 II (Roche). Absolute quantification analysis will be performed with the Lightcycler 480 software (Roche), version 1.5, following the manufacturer's recommendations.

Markers such as LPS binding protein (LBP) and soluble cluster of differentiation 14 (sCD14) will be used as biomarkers to indicate indirect microbiota translocation and gut inflammation. The Intestinal fatty acid binding protein (I-FABP) will be measured to study intestinal permeability. LBP, sCD14, and I-FABP will be evaluated using an Enzyme-Linked ImmunoSorbent Assay (ELISA) in accordance with the manufacturer’s recommendations. The LBP plasma level will be measured using the LBP Soluble ELISA kit (Enzo Life Sciences, Villeurbanne, France), the sCD14 plasma level using the soluble CD14 (human) ELISA kit (Enzo Life Sciences, Villeurbanne, France), and the I-FABP plasma level using the Human I-FABP ELISA kit (Hycult Biotech, Uden, the Netherlands).

### Biobanking

Samples collected as part of the research will constitute a plasma, platelets, CTC and PBMC library to meet the objective of "Constituting a unique *LB* biobank for PDAC to optimize biomolecular knowledge on PDAC at different stages” under the responsibility of the coordinating investigator (Prof Catherine Alix-Panabières). This collection is made in accordance with the current regulations and will be declared to the regulatory authorities. Samples will be stored at the LCCRH-Liquid Biopsy laboratory, an approved laboratory, at—80 °C and liquid nitrogen for plasma and cells, respectively.

### Statistical analysis

In this project, the first type of machine learning model we will train is random forests. These will be used to predict whether a patient will develop pancreatic cancer based on all measured markers and associated health data. The dataset collected in this project will be divided into three different subsets: a training set (60% of the samples), a test set (20% of the samples), and a validation set (20% of the samples). A stratified sampling approach will be used to ensure that each class is accurately represented in each set, especially if an imbalance between classes is present in the original dataset. The training and test subsets will be used to train the machine learning algorithms, while the validation subset will be used to ensure that the model is generalizable (i.e., it can accurately classify future samples that may be collected). We will use the out-of-bag error method (a bootstrapping procedure that trains the models on multiple subsets of the predictor variables) to quantify the relative contribution of each variable to the model's performance. In addition, the performance of each model will be quantified by calculating its accuracy, precision, and area under the curve (AUC). In the performance of the random forest models is not satisfactory, the same approach will be used, but this time using XGboost and artificial neural network models instead. This approach allows the complexity of the model to be gradually increased, with the aim of forming a parsimonious model with a good ability to distinguish between healthy and cancer patients. The performance of all models trained during this project will be compared to a multiple logistic regression model as a baseline model.

We will also explore the results of machine learning models in the inferential framework. Using generalized linear models, we will compare the mean of each marker retained by the best machine learning models, while controlling for confounding variables (e.g., age…). This approach will quantify the differences in markers between healthy and cancer patients and identify markers that may not be statistically significantly different between the two groups but still contribute to good machine learning performance (those variables are called "weak predictors").

Indeed, in some cases, the combination of several "weak predictors" with a machine learning model can lead to good prediction performance. This will provide valuable information on the number and type of markers to be retained for future diagnosis.

#### Endpoints

- Measurements of CTCs, ctDNA, cfnucleosomes, circulating proteins, EVs, CIC, and circulating microbiota as defined above.
- Area Under the Curve (AUC) of the ROC curve of the multi-marker *LB* signature. Optimal threshold of the multi-marker *LB* signature that maximizes the Youden index.
- Dichotomized test at the optimal threshold: positive test (patients with resectable PDAC) if the *LB* signature is greater than or equal to the optimal threshold/negative test (HC) if the *LB* signature is lower than the optimal threshold.
- *Sensitivity*: the percentage of patients with resectable PDAC (gold standard = cytologically and/or histologically proven PDAC) with a positive LB signature test.
- *Specificity*: the percentage of HC with a negative LB signature test.

#### Data management

The database is managed by the Montpellier Cancer Institute Data Center using the Clinsight® software. A data-management plan and a consistency check program were established during the database’s creation. Each investigator has unique usernames and passwords to access the database. A specific and secure login was established to database monitoring. Quality control of the data is conducted in four ways: *i)* automatic data consistency checks; *ii)* data management control through regular queries submissions; *iii)* regular e-control of entered data by the project manager; *iv)* on-site data monitoring of at least 10% of the entered data. A fill rate analysis plan is also regularly sent to the BACAP 2 coordinator by the data manager.

#### Highlights of the PANLIPSY project

- *Patient recruitment*: *(i)* PANLIPSY relies on the well-established and nationally organized BACAP 2 network; *(ii)* Patients presenting all stages of PDAC and benign pancreatic conditions (PACTOL clinical trial) are included to define the blood signature that can detect PDAC at a very early stage;
- *Controls:* Healthy donors will be recruited at the end of each phases DISCOVERY & VALIDATION PHASES to be exactly age- and sex-matched;
- *Blood samples*: A significant volume of blood (50 mL) will be prospectively taken in dedicated tubes to analyze seven circulating biomarkers simultaneously in the same patient;
- *Data quality*: Clinical, radiological and biological data defined by the BACAP cohort will be considered in our signature on top of the *liquid biopsy*;
- *Artificial Intelligence*: At the end of the first DISCOVERY PHASE, AI-based machine learning will combine several pieces of information to identify the blood signature, which can discriminate patients developing pancreatic cancer from healthy donors and those with a benign pancreatic disease. In the VALIDATION PHASES, the *liquid biopsy* will be blindly analyzed with the newly discovered signature to validate it.
- *Feasibility:* The various PANLIPSY partners have already worked together successfully, even publishing a few articles [18–20]. All the technologies needed to achieve our objectives are optimized, robust and implemented daily in the various laboratories.

## Discussion

The growing global prevalence of PC, which is typically detected at an advanced stage due to a lack of a biological test, compelled us to develop an effective screening method for PC detection. In its aim, our consortium consists of four academic institutions with an international track record of research on *LB* (e.g., CTCs, ctDNA, EVs), circulating microbiota and AI-based algorithms. The recruitment of patients with PDAC, pancreatic benign conditions, and HCs is under the supervision of Pr Barbara Bournet (BACAP consortium) and INOVIE Biobank (Christophe Béna), respectively. Since 2015, more than 1,600 patients have been included in the BACAP cohort, with clinical, radiological, epidemiological, and social data linked to the biological samples readily available. In the last year, 100 patients were primarily included from three centers (University Hospital of Toulouse, Cancer Institute of Montpellier, and University Hospital of Lille). These patients represent 25 with resectable, 35 with borderline or locally advanced, and 40 with metastatic PDAC. Based on these inclusion data, we plan to include 215 patients with PDAC at diagnosis and before any treatment, as previously explained. Blood samples from age- and sex-matched HCs will be graciously provided by INOVIE Biobank at the end of each inclusion period.

The area of competences of our network includes: *1/ LB* platforms: *a)* the LCCRH & Liquid Biopsy lab for CTCs detection, CIC evaluation to assess the immune system status and EVs analysis in collaboration with Dr Costa-Silva from Lisbon under the expertise of Pr Alix-Panabières, *b)* Dr Valerie Taly’ lab for ctDNA analysis using universal methylation markers by sensitive ddPCR at the single-fragment ctDNA level, *c)* Pr Jean-Philippe Lavigne and Dr Catherine Dunyach-Remy’ lab for circulating microbiota as a completely new circulating biomarker in LB, *d)* Industrial partner services supervised by Pr Alix-Panabières’ lab for circulating cell-free nucleosomes (VOLITION) and circulating proteins (O-link) analysis; *2/* AI-based algorithmic analyses (after the discovery and validation phases) by Dr Antoine Dujon at the CREEC/CANECEV, MIVEGEC (CREES), University of Montpellier, CNRS, IRD, Montpellier, and statistical analyses by Dr Caroline Mollevi (University Hospital Center of Montpellier, Biostatistics Department) for the calculation of the number of patients and HCs to recruit in this project.

Each partner has substantial expertise in their field of research, as documented by publications in high-ranking journals of basic science and laboratory medicine and by some patents, which demonstrates the translational character of the studies carried out by PIs of this network. Besides the partners’ expertise, the assays tested in PANLIPSY are complementary and allow the detection of different *LB* biomarkers. The presented project will provide unique information on the assay combination(s) with the strongest clinical relevance for early PDAC detection. Thus, the partners’ expertise is complementary and there is a clear added value of performing this prospective multi-biomarker validation study.

Moreover, we are taking advantage of this project to set up a unique biobank dedicated to liquid biopsy in the PDAC for any new biomarkers discovered in the coming years.

## Data Availability

No datasets were generated or analysed during the current study.

## Abbreviations

BACAP: French biological and clinical database for PDAC cohort
BPC: Benign pancreatic conditions
CA 19-9: Carbohydrate antigen 19–9
CIC: Circulating immune cell
CTC: Circulating tumor cell
ccfDNA: Circulating cell free DNA
cfnucleosomes: Circulating cell-free nucleosomes
ctDNA: Circulating cell-free tumor DNA
ddPCR: Digital droplet PCR
ELBS: European liquid biopsy society
EV: Extracellular vesicles
HCs: Healthy controls
LB: Liquid biopsy
LCCRH: Laboratory of rare circulating human cells
MRI: Magnetic resonance imaging
PACTOL: Pancreatic disease cohort of Toulouse
PAR: Patients at risk
PBMC: Peripheral blood mononuclear cells
PBS: Phosphate-buffered saline
PC: Pancreatic cancer
PPNL: Pre-neoplastic cystic lesion of the pancreas
PDAC: Pancreatic ductal adenocarcinoma

## References

[1] Pereira SP (2020). Early detection of pancreatic cancer. Lancet Gastroenterol Hepatol.

[2] Goggins M (2005). Molecular markers of early pancreatic cancer. J Clin Oncol.

[3] Conroy T, et al. Pancreatic cancer: ESMO Clinical Practice Guideline for diagnosis, treatment and follow-up. Ann Oncol. 2023;34(11):987–1002. 10.1016/j.annonc.2023.08.009 37678671

[4] Pantel K, Alix-Panabières C (2010). Circulating tumour cells in cancer patients: challenges and perspectives. Trends Mol Med.

[5] Alix-Panabières C, Pantel K (2021). Liquid biopsy: from discovery to clinical application. Cancer Discov.

[6] Wu H (2022). Noninvasive detection of pancreatic ductal adenocarcinoma using the methylation signature of circulating tumour DNA. BMC Med.

[7] Nitschke C (2023). Peripheral and portal venous KRAS ctDNA detection as independent prognostic markers of early tumor recurrence in pancreatic ductal adenocarcinoma. Clin Chem.

[8] Duffy MJ, Diamandis EP, Crown J (2021). Circulating tumor DNA (ctDNA) as a pan-cancer screening test: is it finally on the horizon?. Clin Chem Lab Med.

[9] Pons-Belda OD, Fernandez-Uriarte A, Diamandis EP (2021). Can circulating tumor DNA support a successful screening test for early cancer detection? The Grail paradigm. Diagnostics (Basel).

[10] Alix-Panabières C (2020). The future of liquid biopsy. Nature.

[11] Canivet C (2018). A prospective clinical and biological database for pancreatic adenocarcinoma: the BACAP cohort. BMC Cancer.

[12] Pietrasz D (2022). Prognostic value of circulating tumour DNA in metastatic pancreatic cancer patients: post-hoc analyses of two clinical trials. Br J Cancer.

[13] Garrigou S (2016). A study of hypermethylated circulating tumor DNA as a universal colorectal cancer biomarker. Clin Chem.

[14] Huggett JF (2020). The digital MIQE guidelines update: minimum information for publication of quantitative digital PCR experiments for 2020. Clin Chem.

[15] Holdenrieder S (2014). Novel serum nucleosomics biomarkers for the detection of colorectal cancer. Anticancer Res.

[16] Van den Ackerveken P (2023). Epigenetic profiles of elevated cell free circulating H3. 1 nucleosomes as potential biomarkers for non-Hodgkin lymphoma. Sci Rep.

[17] Kramski M (2011). Novel sensitive real-time PCR for quantification of bacterial 16S rRNA genes in plasma of HIV-infected patients as a marker for microbial translocation. J Clin Microbiol..

[18] Buscail E (2019). High clinical value of liquid biopsy to detect circulating tumor cells and tumor exosomes in pancreatic ductal adenocarcinoma patients eligible for up-front surgery. Cancers.

[19] Cortés-Hernández LE (2021). Current applications and discoveries related to the membrane components of circulating tumor cells and extracellular vesicles. Cells.

[20] Rifai N. *Tietz Textbook of Laboratory Medicine-E-Book: Tietz Textbook of Laboratory Medicine-E-Book*. England: Elsevier Health Sciences; 2022.

